# Isolation, Identification, and Genomic Characterization of a Cellulolytic *Bacillus subtilis* A2 from Goose Ileum

**DOI:** 10.3390/microorganisms14061272

**Published:** 2026-06-05

**Authors:** Linghong Sun, Zhengkun Chen, Yvqing Peng, Shoubao Yan

**Affiliations:** 1 Department of Biological Engineering, Huainan Normal University, Huainan 232038, China; sunlinghong1310@163.com (L.S.); 15256547504@163.com (Z.C.); 13065847663@163.com (Y.P.); 2 Anhui Engineering Research Center for Development and Application of Brewing Industry Microbial Resource, Huainan Normal University, Huainan 232038, China

**Keywords:** *Bacillus subtilis* A2, cellulose degradation, cellulase, enzymatic properties, whole-genome sequencing

## Abstract

To identify efficient cellulose-degrading microbes suitable for the animal intestinal environment and to address the low utilization of crude fiber in feed, eight cellulolytic strains were isolated from the ileum of Yangzhou geese. Among them, strain A2 showed the highest cellulolytic activity (D/d = 1.48) via the CMC (carboxymethyl cellulose) agar transparent zone method. Based on whole-genome-based identification, strain A2 was identified as *Bacillus subtilis*. Whole-genome sequencing revealed a circular chromosome of 4.02 Mb with a GC content of 43.72%, containing 4083 protein-coding sequences, of which 7.40% were involved in carbohydrate transport and metabolism. CAZyme annotation identified 167 carbohydrate-active enzyme genes, including 64 glycoside hydrolase genes, along with 60 hemicellulase and 3 lignin-degrading enzyme genes, forming a complete lignocellulose-degrading system. The cellulase from A2 exhibited optimal activity at 55 °C and pH 7.0, with good stability at 50–65 °C and pH 5–7, and was significantly inhibited by Cu^2+^, Mn^2+^, and Zn^2+^. Notably, its degradation efficiency toward microcrystalline cellulose reached 197% of that toward CMC. In conclusion, *B. subtilis* A2, with its excellent enzymatic properties and robust genetic foundation, is a promising candidate for developing feed enzymes and enhancing lignocellulose utilization.

## 1. Introduction

Cellulose is the most abundant renewable organic resource in nature. As the primary structural component of plant cell walls, it constitutes 35–50% of plant dry matter [1]. However, its efficient degradation is hindered by its complex crystalline structure and the dense network it forms with hemicellulose and lignin, posing a major bottleneck in the resource utilization of agricultural waste [2]. Enzymatic hydrolysis, valued for its environmental friendliness and mild reaction conditions, is considered a crucial approach for achieving efficient cellulose conversion. The foundation of this approach lies in screening and obtaining elite microbial strains with high cellulase-producing capabilities.

Cellulase is a multi-enzyme complex system that specifically hydrolyzes the β-1,4 glycosidic bonds of cellulose. It primarily comprises three components: endoglucanase (EC 3.2.1.4), exoglucanase (EC 3.2.1.91), and β-glucosidase (EC 3.2.1.21) [3]. Among these, endoglucanase acts on the amorphous regions within the cellulose chain, randomly cleaving glycosidic bonds to produce oligosaccharides of varying lengths, thereby initiating the cellulose degradation process. Cellulases are widely distributed among microorganisms, including bacteria, fungi, and actinomycetes. The *Bacillus*, as an important industrial enzyme producer, has garnered significant attention in cellulase research due to its rapid growth, short enzyme production cycle, ease of enzyme protein purification, and the fact that most species are non-pathogenic [4].

The digestive tract of poultry harbors abundant microbial resources, and the intestinal environment has evolved over time to form a unique microbial community structure adapted to the host’s digestive characteristics. Geese, as typical herbivorous poultry, possess a strong capacity for crude fiber digestion, and their intestinal microorganisms play a crucial role in the cellulose degradation process [5]. Research indicates that the cecum and ileum of geese contain a large number of bacteria with cellulolytic activity, including genera such as *Bacillus*, *Lactobacillus*, and *Enterococcus* [5,6]. Isolating endogenous cellulose-degrading bacteria from the goose intestine not only helps elucidate the microbiological mechanisms of cellulose digestion in poultry but also provides excellent microbial strain resources for the development of novel feed enzymes [6].

With the rapid advancement of high-throughput sequencing technologies, whole-genome sequencing has become a powerful tool for dissecting the functional genes of microorganisms. By performing genome sequencing and functional annotation of cellulose-degrading bacteria, the genetic basis underlying their cellulase production can be elucidated at the molecular level. The Carbohydrate-Active enzymes (CAZymes) database specifically catalogues enzyme families involved in the degradation, modification, and biosynthesis of carbohydrates, including glycoside hydrolases (GHs), glycosyltransferases (GTs), polysaccharide lyases (PLs), carbohydrate esterases (CEs), and auxiliary modules (AAs) [7]. Integrated annotation using multiple databases such as COG, GO, and KEGG enables a comprehensive analysis of the strain’s carbohydrate metabolic pathways and lignocellulolytic enzyme systems, providing a genomic basis for a deeper understanding of its degradation mechanisms [8].

In this study, bacterial strains with cellulose-degrading capability were isolated from the goose ileum. The high-yielding strains obtained through screening were subjected to morphological, physiological, biochemical, and molecular biological identification, and the enzymatic properties of their cellulase production were systematically investigated. On this basis, whole-genome sequencing and assembly were performed on strain A2, identified as *Bacillus subtilis*. The genes involved in carbohydrate metabolism were analyzed through COG, GO, and KEGG database annotations, while carbohydrate-active enzymes were identified using CAZymes database annotation to elucidate the genetic basis of its cellulose degradation capability. The findings of this study will provide a theoretical foundation for revealing the molecular mechanisms of goose-derived cellulose-degrading bacteria and lay the groundwork for developing novel feed cellulase preparations and promoting the resource utilization of agricultural waste.

## 2. Materials and Methods

### 2.1. Experimental Materials

Experimental Animals: Healthy 360-day-old Yangzhou geese.

Screening Medium: 5.0 g CMC-Na (sodium carboxymethyl cellulose) and 18.0 g agar were added to approximately 900 mL of distilled water. The mixture was heated to boiling with continuous stirring until the CMC-Na and agar were completely dissolved. Then, 1.0 g K_2_HPO_4_, 20.0 g (NH_4_)_2_SO_4_, 0.5 g NaCl, and 0.5 g MgSO_4_ were added and dissolved. The solution was then adjusted to a final volume of 1000 mL with distilled water to compensate for water loss due to evaporation. The pH was unadjusted (natural pH). The medium was sterilized by autoclaving at 121 °C for 20 min.

Fermentation Enzyme Production Medium: 20 g of wheat bran, 20 g of soybean meal (Anhui Xinkang Feed Co., Ltd., Hefei, Anhui, China), 5 g of CMC-Na, and 5 g of NaCl were mixed and brought to approximately 900 mL with distilled water. The mixture was thoroughly stirred and heated to dissolve the CMC-Na. After cooling, the volume was adjusted to 1000 mL with distilled water to compensate for evaporation. The pH was unadjusted (natural pH). The medium was autoclaved at 121 °C for 20 min.

Seed Culture Medium: 10 g of peptone (Sangon Biotech (Shanghai) Co., Ltd., Shanghai, China), 5 g of yeast extract powder (Sangon Biotech (Shanghai) Co., Ltd., Shanghai, China), and 5 g of NaCl were dissolved in approximately 900 mL of distilled water. If necessary, gentle heating was applied to ensure complete dissolution. The volume was then adjusted to 1000 mL with distilled water. The pH was unadjusted (natural pH). The medium was autoclaved at 121 °C for 20 min.

### 2.2. Sample Collection

Six healthy 360-day-old Yangzhou geese were randomly selected from a commercial farm (Changzhou Sijì Poultry Industry Co., Ltd., Changzhou, Jiangsu Province, China), with half males and half females. After fasting for 24 h, the geese were sacrificed by cervical dislocation and dissected. Both ends of the ileum were ligated with string and then cut from the outside of the ligations. The surface of the ileum was disinfected with 75% alcohol. Subsequently, the ileum was cut open, and the mucus from the intestinal wall was scraped into 50 mL of sterile water. The mixture was homogenized and set aside for further use.

### 2.3. Screening of Cellulase-Producing Strains

Primary Screening: The ileal fluid samples were serially diluted. An aliquot of 0.1 mL from each dilution was spread onto screening medium and incubated at 37 °C for 48 h. Well-growing single colonies were marked. These colonies were repeatedly streaked onto fresh CMC medium for isolation and purification. The purified strains were preserved for future use.

Secondary Screening: The strains obtained from the primary screening were inoculated onto screening medium using the spot inoculation method. After incubation at 37 °C for 48 h, the plates were stained with 10 mg/mL Congo red dye for 30 min. The diameter of the hydrolysis zone (D) and the diameter of the colony (d) were measured. The D/d ratio was calculated to screen for strains with strong cellulose-degrading ability.

### 2.4. Whole Genome Sequencing and Genome Feature Prediction

Genomic DNA of the bacteria was extracted using a DNA extraction kit (Beyotime, Catalog No. R0017S, Shanghai, China). The quality and concentration of the extracted DNA were determined using 1% agarose gel electrophoresis and a nucleic acid quantifier. After passing quality inspection, the samples were sent to General Biosystems Co., Ltd. (Chuzhou, China) for sequencing. This project employed the Whole Genome Shotgun (WGS) sequencing strategy to construct libraries with different insertion fragments. Using Next-Generation Sequencing (NGS) technology based on the Illumina NovaSeq 6000 (Illumina, Inc., San Diego, CA, USA) sequencing platform, paired-end (PE) sequencing was performed on the libraries. Libraries were constructed from the samples, and second-generation sequencing data were obtained. Fastp v0.23.4 software was used for quality control of the sequencing data. SPAdes v3.15.5 software was used for de novo assembly of the second-generation data. Scaffolds with a length greater than 500 bp and an average depth greater than 10 were selected as the assembly results to construct scaffolds and contigs. Pilon v1.24 software was used for single-base correction. Finally, the contigs were spliced to obtain a complete sequence.

tRNA genes in the whole genome were predicted using tRNAscan-SE v2.0, and rRNA genes were predicted using Barrnap v0.9. Predictions for other non-coding RNAs were primarily obtained by comparison with the Rfam database (https://rfam.org/ (accessed on 28 April 2025)). GeneMarkS v4.17 software was used to predict protein-coding genes in the bacterial genome. CRISPRCasFinder v4.3.6 software was used to predict CRISPRs in the whole genome. PhiSpy v4.2.21 was used to predict prophages present in the genome. Circos v0.69-9 software was used to generate a circular map of the top 20 scaffolds with sequence lengths greater than 5000 bp in the sample.

### 2.5. Identification of Cellulase-Producing Strains

Morphological Observation of Strains: The screened strains were inoculated onto the screening medium using the quadrant streak method to obtain isolated colonies. They were cultured overnight at 37 °C until distinct single colonies were observed. The morphology of the colonies and the bacterial lawn was observed, and Gram staining was performed.

Identification of Strain A2 Based on Whole-Genome Sequencing: For accurate taxonomic identification, genome-based analyses were conducted using the Type (Strain) Genome Server (TYGS, https://tygs.dsmz.de/ (accessed on 18 May 2026)) and the EzBioCloud platform (https://www.ezbiocloud.net/ (accessed on 18 May 2026)). The OrthoANIu value between strain A2 and closely related reference strains was calculated using the ANI calculator available on the EzBioCloud website. The dDDH value was estimated using the TYGS platform, which employs the Genome-to-Genome Distance Calculator (GGDC). A phylogenetic tree was automatically constructed by TYGS based on genome-wide sequence similarity, and the tree was visualized and annotated using iTOL. According to established thresholds, species demarcation was defined as OrthoANIu ≥ 95–96% and dDDH ≥ 70%.

### 2.6. Functional Annotation of Protein-Coding Genes

NR annotation of protein-coding genes (CDS) was performed using diamond software. The gene-encoded protein sequences were aligned with protein sequences in the NR database (https://ftp.ncbi.nlm.nih.gov/blast/db/FASTA/ (accessed on 30 April 2025)), with alignment parameters set to an E-value of 1 × 10^−6^, selecting the best hit. Eggnog-mapper v2.1.12 software was used to predict protein-coding genes. Based on the GO IDs from the InterProScan v5.65-97.0 prediction results, GOSlim annotation was performed with map2slim (from go-perl package, v0.15). KEGG (Kyoto Encyclopedia of Genes and Genomes) annotation of protein-coding genes was primarily completed using KEGG’s KAAS automated annotation system. Hmmscan v3.3.2 software was used to align the sample’s gene-encoded protein sequences with the local CAZymes database (http://www.cazy.org/ (accessed on 30 April 2025)) to predict the presence of CAZymes genes in the genome sequence.

### 2.7. Determination of Enzymatic Properties

The single-factor method was employed to investigate the effects of different temperatures (30 °C, 35 °C, 40 °C, 45 °C, 50 °C, 55 °C, 60 °C, 65 °C, 70 °C, and 75 °C), different pH values (3.0, 4.0, 5.0, 6.0, 7.0, 8.0, and 9.0), and different metal ions (1 mmol/L Cu^2+^, Fe^3+^, K^+^, Zn^2+^, Mn^2+^, and Fe^2+^) on the cellulase activity of the target strains. The stability of the target strains’ cellulase was assessed at different temperatures (50 °C, 55 °C, 60 °C, 65 °C, and 70 °C) and different pH values (3.0, 4.0, 5.0, 6.0, 7.0, 8.0, and 9.0). Additionally, the substrate specificity of the cellulase was evaluated using different substrates, including CMC-Na, absorbent cotton, dried soybean residue, filter paper, wheat bran, and microcrystalline cellulose. The activity of cellulase was determined using the DNS method. For the determination of enzymatic properties, the strain was cultured in fermentation enzyme production medium. After incubation, the culture broth was centrifuged at 8000× *g* for 10 min at 4 °C to obtain the crude extracellular enzyme supernatant, which was used as the enzyme source for all subsequent assays, including temperature, pH, metal ion, and substrate specificity tests. All experiments for enzymatic property determination, including temperature, pH, metal ion effects, and substrate specificity tests, were performed in triplicate (*n* = 3) and repeated independently three times. Data are presented as the mean ± standard deviation (SD) from three independent experiments.

### 2.8. Statistics Analysis

IBM SPSS Statistics 26.0 software was used to perform one-way ANOVA followed by LSD post hoc test. This analysis was conducted to compare the D/d among different strains, as well as to evaluate the effects of various metal ions on enzyme activity and the substrate specificity of the cellulase. The data were considered significant at *p* < 0.05.

## 3. Results

### 3.1. Screening of Cellulose-Degrading Strains

**Figure 1:**
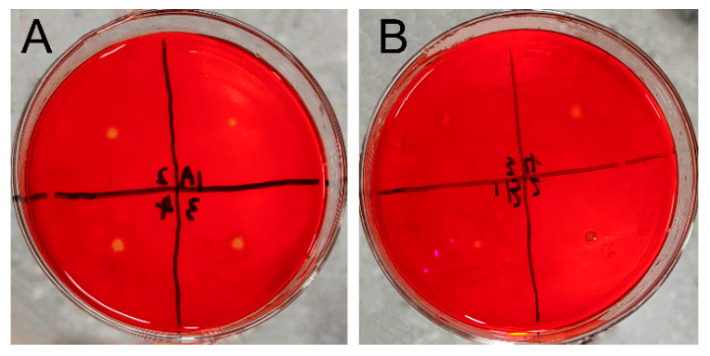
Cellulose-degrading activity of isolated strains on CMC agar plates. (**A**) Strains A1, A2, A3, and A4; (**B**) Strains B1, B2, B3, and B4.

In this study, eight bacterial strains (A1, A2, A3, A4, B1, B2, B3, and B4) were isolated and purified from the ileal mucosa of Yangzhou geese using CMC agar medium. Among the eight purified strains, strain A2 exhibited the highest D/d (D: 3.44 mm, d: 2.32 mm, D/d: 1.48), demonstrating the strongest cellulose hydrolysis ability (Figure 1 and Table 1).

### 3.2. Complete Genome Information of Strain A2

**Figure 2:**
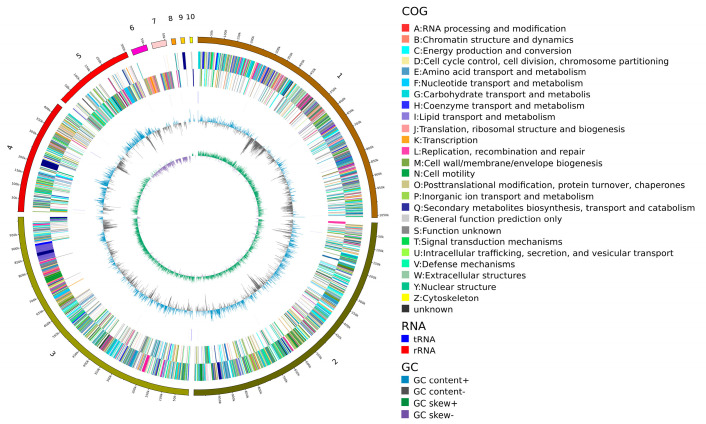
Whole genome map of strain A2. From the outside to the inside: chromosome karyotype; CDS on the positive strand and negative strand (different colors represent different COG functional classifications of CDS); tRNA and rRNA; GC content; the innermost circle represents GC skew.

The genome of strain A2 consists of a single circular chromosome with a total length of 4,021,044 bp and an average GC content of 43.72% (Figure 2 and Table 2). It contains 4083 CDS, accounting for 88.56% of the total genome length, along with 93 non-coding RNAs (ncRNAs), 59 transfer RNAs (tRNAs), 18 prophages, and 1 CRISPRs.

### 3.3. Identification of Strain A2

**Figure 3:**
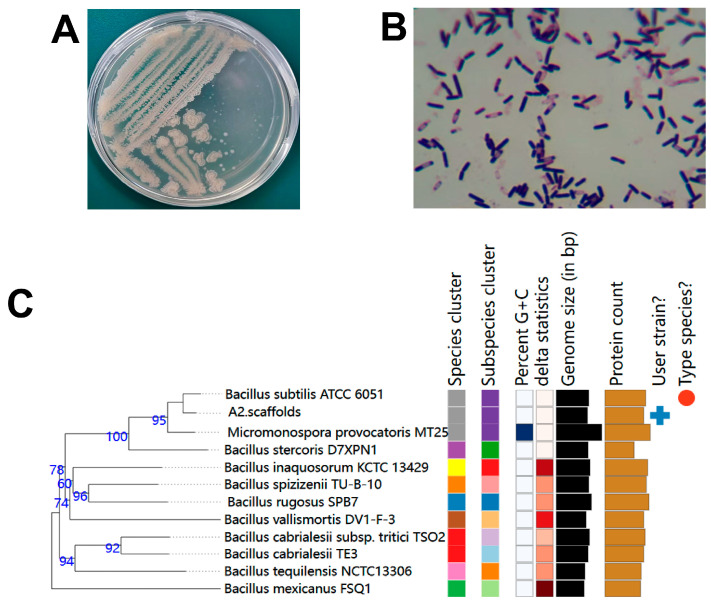
Identification of cellulose-degrading strain A2. (**A**) Colony morphology of the strain A2; (**B**) Gram staining of strain A2 (observed under 1000× magnification); (**C**) Genome-based phylogenetic tree of strain A2.

The colonies of strain A2 appeared light brown, large, and flat with irregular edges. The surface of both the colonies and the bacterial lawn appeared dry (Figure 3A). Gram staining and microscopic examination revealed that strain A2 was a Gram-positive (blue-purple), rod-shaped bacterium capable of producing spores (Figure 3B). The OrthoANIu value between strain A2 and *Bacillus subtilis* ATCC 6051 (GCF_000009045.1) was 99.06%, and the dDDH value was 92%, both significantly exceeding the established species thresholds (95–96% for OrthoANIu and 70% for dDDH). In contrast, the OrthoANIu and dDDH values between strain A2 and other closely related *Bacillus* species were below the species demarcation thresholds (Table 3). Phylogenetic analysis based on core genome sequences further confirmed that strain A2 formed a well-supported clade with *B. subtilis* reference strains (Figure 3C). Collectively, these genome-based analyses unequivocally identified strain A2 as *Bacillus subtilis*, and which was consequently named *Bacillus subtilis* A2.

### 3.4. Gene Function Annotation

**Figure 4:**
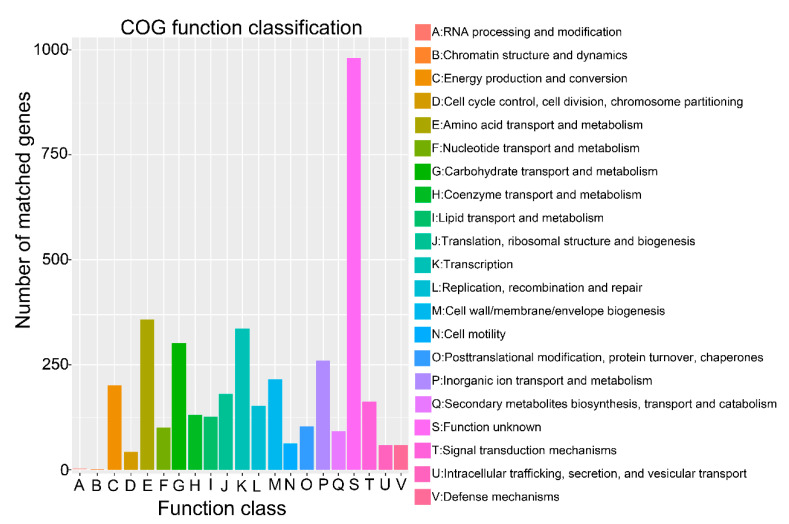
COG functional classification of *B. subtilis* A2 genome.

In the eggNOG database annotation (Figure 4), the 4083 CDSs of *B. subtilis* A2 were annotated into 25 COG categories, with 3921 genes assigned to 21 meaningful COG categories (Supplementary Table S1 and Figure 4). Among these sequences, 981 genes were classified as “Function unknown” requiring further investigation. Among the genes with known functional annotations, the largest number were assigned to “Amino acid transport and metabolism” (358, 8.77%), followed by “Transcription” (336, 8.23%), “Carbohydrate transport and metabolism” (302, 7.40%), “Inorganic ion transport and metabolism” (260, 6.27%), “Cell wall/membrane/envelope biogenesis” (215, 5.27%), and “Energy production and conversion” (201, 4.92%). To elucidate the potential of *B. subtilis* A2 in cellulose degradation at the genetic level, COGs involved in carbohydrate metabolism were further analyzed. These primarily included COG0524 (Sugar kinases, ribokinase family), COG2814 (Major facilitator superfamily), COG1263 (Phosphotransferase system), COG0477 (Major facilitator superfamily), COG1940 (Glucokinase), and COG2723 (Belongs to the glycosyl hydrolase 1 family) (Supplementary Table S2). The high diversity of functional annotations suggests that *B. subtilis* A2 possesses a genetic basis for cellulose degradation.

**Figure 5:**
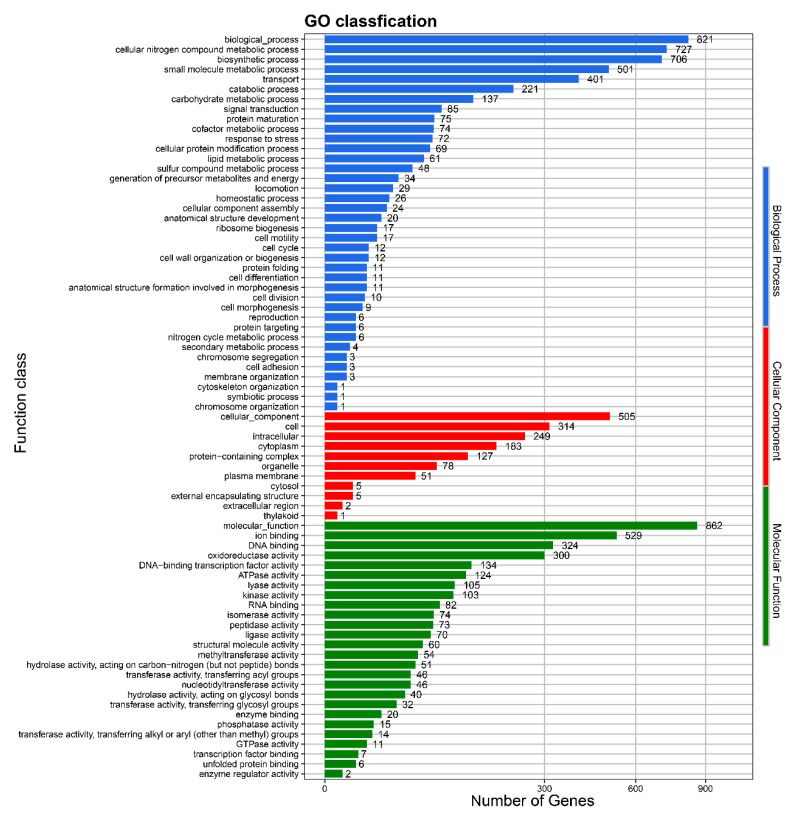
GO annotation of *B. subtilis* A2 genome.

Functional prediction analysis of *B. subtilis* A2 was performed using the GO database, categorizing gene functions into three main groups: biological process, cellular component, and molecular function (Figure 5). GO annotation indicated that the biological process category contained the highest number of genes (4275), followed by molecular function (3184 genes) and cellular component (1520 genes) (Supplementary Table S3, Supplementary Table S4 and Figure 5). Within biological processes, genes were primarily involved in biological_process (GO: 0008150, 821 genes), cellular nitrogen compound metabolic process (GO: 0034641, 727 genes), biosynthetic process (GO: 0009058, 706 genes), small molecule metabolic process (GO: 0044281; 501 genes), and transport (GO: 0006810; 401 genes). Additionally, *B. subtilis* A2 was found to possess diverse cellular components and molecular functions. Regarding molecular function, the five most predominant pathways were molecular_function (GO: 0003674, 862 genes), ion binding (GO: 0043167, 529 genes), DNA binding (GO: 0003677, 324 genes), oxidoreductase activity (GO: 0016491; 300 genes), and DNA-binding transcription factor activity (GO: 0003700; 134 genes). These findings suggest that *B. subtilis* A2 harbors a wide range of molecular functions within its cells. For cellular components, the primary categories were cellular_component (GO: 0005575, 505 genes), cell (GO: 0005623, 314 genes), intracellular (GO: 0005622, 249 genes), cytoplasm (GO: 0005737, 183 genes), and protein-containing complex (GO: 0032991, 127 genes). This indicates the potential of *B. subtilis* A2 to perform various cellular processes within its cells. Furthermore, carbohydrate metabolic process (GO: 0005975, 137 genes) under biological process and hydrolase activity, acting on glycosyl bonds (GO: 0016798, 40 genes) under molecular function were the GO terms directly associated with carbohydrate metabolism.

**Figure 6:**
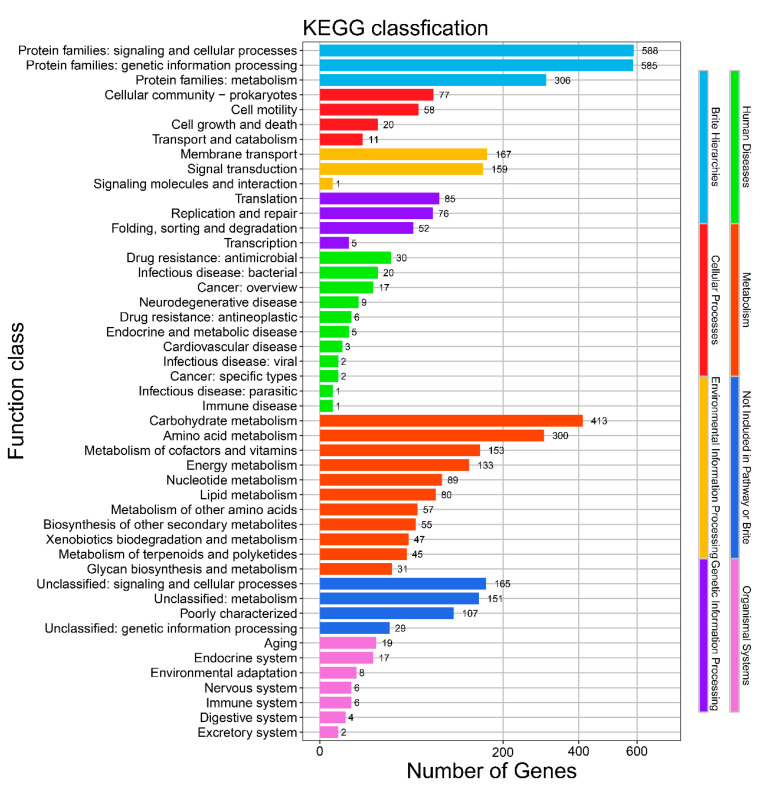
KEGG annotation of *B. subtilis* A2 genome.

A total of 4203 KEGG-annotated genes were identified in the genome of *B. subtilis* A2 (Figure 6). These genes were assigned to six different pathway levels. The distribution of these genes across categories was as follows: 166 genes associated with cellular processes, 327 genes with environmental information processing, 218 genes with genetic information processing, 96 genes with human diseases, 1403 genes with metabolism, and 62 genes with organismal systems. Within the metabolism-related genes, those annotated to carbohydrate metabolism (413), energy metabolism (133), and glycan biosynthesis and metabolism (31) were identified. Among these, pathways such as starch and sucrose metabolism (ko00500, 46 genes), glycolysis/gluconeogenesis (ko00010, 38 genes), pyruvate metabolism (ko00620, 43 genes), and the pentose phosphate pathway (ko00030, 26 genes) were predominant (Supplementary Table S5). These results indicate that *B. subtilis* A2 possesses strong capabilities in carbohydrate and protein metabolism, suggesting its potential value for applications in various industries, including food production and biotechnology.

### 3.5. CAZymes Annotation

**Figure 7:**
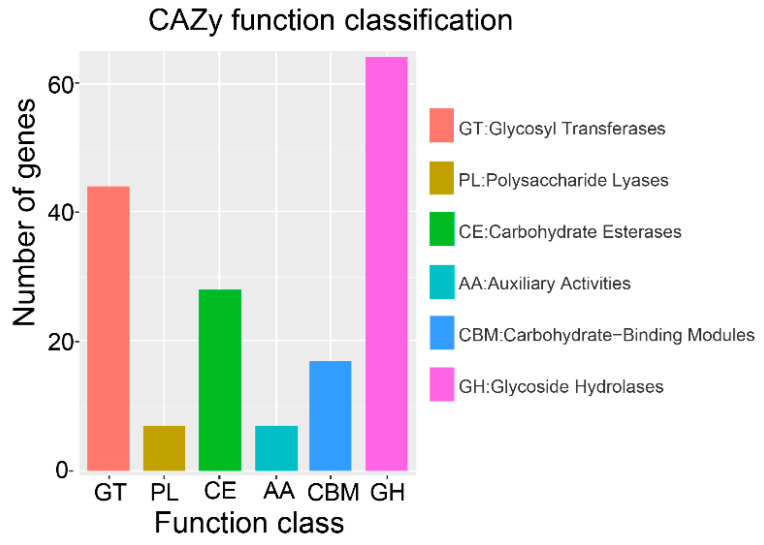
Functional annotation of CAZy databases of *B. subtilis* A2. GH, glycoside hydrolase; GT, glycosyltransferase; PL, polysaccharide lyase; CE, carbohydrate esterase; AA, auxiliary activity enzyme.

The genomic CAZymes annotation results show that 167 CAZyme genes were annotated in the genome of *B. subtilis* A2, accounting for 4.09% of all CDS. Among these, glycoside hydrolases (GHs) play a key role in carbohydrate degradation, with 64 GH genes annotated in the genome (Figure 7). In addition, 44 glycosyl transferase (GT) genes, 28 carbohydrate esterase (CE) genes, 17 carbohydrate-binding module (CBM) genes, 7 polysaccharide lyase (PL) genes, and 7 auxiliary activity (AA) enzyme genes were identified (Supplementary Table S6). A total of 35 GH families were annotated, with GH177, GH4, GH1, GH18, and GH23 being the most abundant, containing 6, 5, 4, 4, and 4 members, respectively. The 28 CE genes were assigned to 8 CE families, including CE1, CE4, CE12, among which the CE1 family had the highest number of genes, with 11 members. Four AA families were annotated, with AA7 being the most abundant. Six PL families were annotated, including PL11 and PL22 (Supplementary Table S6). Among the six CAZy classes, glycoside hydrolases (GHs) exhibited the highest enrichment of related genes, and the cellulase investigated in this study belongs precisely to the glycoside hydrolase class.

### 3.6. Genes Related to Lignocellulolytic Enzymesn

Information on lignocellulose degradation-related enzymes was screened from the CAZy annotation (Table 4).

Regarding cellulolytic enzyme annotation, the *B. subtilis* A2 genome contained five genes annotated as β-glucosidase (EC 3.2.1.21), five genes annotated as exo-β-1,4-glucanase (EC 3.2.1.74), nine genes annotated as 6-phospho-β-glucosidase (EC 3.2.1.86), and one gene annotated as exo-1,3-1,4-glucanase (EC 3.2.1.-). β-Glucosidase (EC 3.2.1.21) and exo-β-1,4-glucanase (EC 3.2.1.74) belong to the GH1 and GH3 families, 6-phospho-β-glucosidase (EC 3.2.1.86) belongs to the GH1 and GH4 families, and exo-1,3-1,4-glucanase (EC 3.2.1.-) belongs to the GH3 family. The abundance of cellulase genes indicates that *B. subtilis* A2 possesses strong cellulose degradation potential.

A total of 60 genes in the *B. subtilis* A2 genome encode hemicellulases, including one endo-β-1,4-xylanase (EC 3.2.1.8) gene, four β-xylosidase (EC 3.2.1.37) genes, one xylan 1,4-β-xylosidase (EC 3.2.1.37) gene, two β-mannanase (EC 3.2.1.78) genes, four β-mannosidase (EC 3.2.1.25) genes, one α-L-arabinofuranosidase (EC 3.2.1.55) gene, five α-glucuronidase (EC 3.2.1.139) genes, five α-galactosidase (EC 3.2.1.22) genes, 11 feruloyl esterase (EC 3.1.1.73) genes, 24 acetyl xylan esterase (EC 3.1.1.72) genes, one endo-β-1,4-galactanase (EC 3.2.1.89) gene, and one exo-1,4-β-xylosidase (EC 3.2.1.-) gene. These hemicellulases belong to 11 families, including GH1, GH3, GH4, GH11, GH26, GH53, CE1, CE3, CE4, CE7, and CE12. The abundance of hemicellulase genes indicates that *B. subtilis* A2 possesses strong hemicellulose degradation potential.

The *B. subtilis* A2 genome contained several lignin degradation-related genes, including one laccase (EC 1.10.3.2) gene, one ferroxidase (EC 1.10.3.-) gene, and one laccase-like multicopper oxidase (EC 1.10.3.-) gene. The proteins encoded by these genes all belong to the AA1 family. This result suggests that *B. subtilis* A2 also has lignin degradation capability.

### 3.7. Enzymatic Properties of the Cellulase

**Figure 8:**
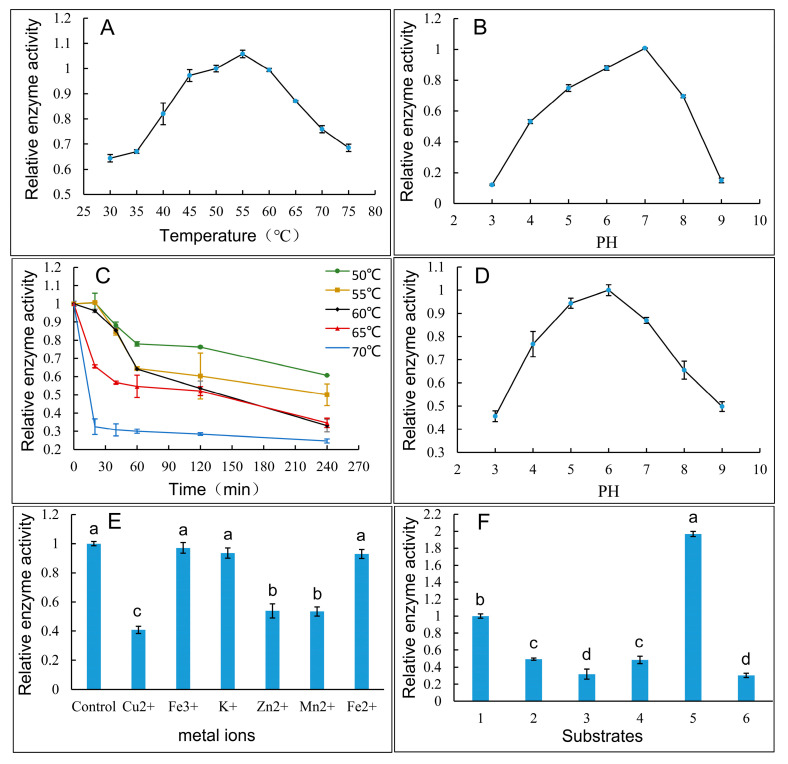
Enzymatic properties of cellulase. (**A**) Effect of temperature on cellulase activity; (**B**) Effect of pH on cellulase activity; (**C**) Thermal stability of cellulase; (**D**) pH stability of cellulase; (**E**) Effect of metal ions on enzyme activity; (**F**) Determination of cellulase activity with different substrates. 1, CMC–Na; 2, absorbent cotton; 3, dried okara (soybean residue); 4, wheat bran; 5, microcrystalline cellulose; 6, filter paper. ^a–d^ indicate significant differences between groups not sharing the same superscript (*p* < 0.05). *n* = 3.

The optimum temperature and pH for the cellulase produced by strain A2 were determined to be 55 °C and pH 7.0, respectively (Figure 8A,B). Stability assays revealed that the enzyme exhibited good thermal stability between 50 °C and 65 °C (Figure 8C). Furthermore, it maintained high stability after incubation at 30 °C for 18 h over a pH range of 5.0–7.0 (Figure 8D).

The effects of various metal ions at a concentration of 1 mmol/L on cellulase activity were also investigated. The results showed that Cu^2+^, Mn^2+^, and Zn^2+^ significantly inhibited enzyme activity (Figure 8E, *p* < 0.05). Among these, Cu^2+^ exhibited the strongest inhibitory effect, reducing activity by 59.2%. This inhibition might be attributed to metal chelation disrupting the enzyme’s active center.

Substrate specificity tests demonstrated that the enzyme exhibited the highest degradation efficiency towards microcrystalline cellulose (relative activity of 197%), followed by absorbent cotton (49.2%) and wheat bran (48.4%). In contrast, it showed relatively lower activity against filter paper (30.5%) and dried soybean residue (32.0%) (Figure 8F, *p* < 0.05).

## 4. Discussion

### 4.1. Screening and Identification of Cellulose-Degrading Strains: Host Specificity and Taxonomic Significance

The screening of cellulose-degrading bacteria is a core prerequisite for the development of lignocellulose conversion technology, and the selection of isolation sources directly determines the functional suitability of the strains. Currently, isolation sources for cellulose-degrading bacteria are predominantly concentrated in environments such as soil, compost, and humus [9,10], while research on isolating efficient cellulose-degrading bacteria from poultry intestinal mucosa remains relatively scarce. In this study, eight strains of cellulose-degrading bacteria were isolated from the ileal mucosa of Yangzhou geese. Among them, *B. subtilis* A2 exhibited a D/d value of 1.48. This finding not only reveals that the poultry intestinal microecosystem harbors underutilized resources of efficient cellulose-degrading bacteria but also validates the effectiveness of the “functionally adaptive habitat screening” strategy.

As a locally renowned herbivorous waterfowl breed in China, Yangzhou geese are fed a diet with a crude fiber content as high as 18–22%. Through long-term adaptive evolution, the ileal mucosa has developed a microecological community centered around cellulose-degrading bacteria. Specifically, the mucus layer on the ileal mucosal surface provides a stable attachment site for these strains, while the continuous nutritional competition within the intestinal tract drives the evolution of highly efficient cellulose hydrolysis capabilities. This unique niche provides dual assurance for the high activity of strain A2 [11,12].

A key advantage of this strain is the presence of a spore structure. Spores possess strong resistance to extreme environments, including high temperatures, drought, acid-base stress, and chemical disinfectants. This characteristic enables the strain to maintain high survival rates during industrial fermentation and ensures its colonization and functional efficacy as a feed additive within the animal gut, significantly enhancing its practical application value [13,14].

### 4.2. Genomic Features and Functional Annotation: Deciphering the Genetic Basis of Cellulose Degradation

The whole-genome sequencing of *B. subtilis* A2 provides comprehensive data support for deciphering the genetic basis of its cellulose-degrading capability. The genome has a total length of 4,021,044 bp, a GC content of 43.72%, and encodes 4083 CDS. These genomic characteristics are largely consistent with those of reported *B. subtilis* model strains (e.g., *B. subtilis* 168, with a genome length of 4,215,606 bp and GC content of 43.5%), indicating that its genetic background is typical and conserved [15,16]. The presence of a CRISPRs system suggests that this strain possesses a certain capacity for phage defense, enabling it to maintain genomic stability in complex environments. The presence of prophages may provide pathways for horizontal gene transfer, facilitating the strain’s acquisition of exogenous genes that enhance environmental adaptability and functional diversity [17,18].

Annotation using the eggNOG database revealed that genes related to carbohydrate transport and metabolism (COG category G) account for 7.40% of the total. This high proportion of genes associated with carbohydrate transport and metabolism provides genetic evidence confirming the strain’s robust capacity for carbohydrate degradation and utilization. Further analysis indicated that the COG categories involved in carbohydrate metabolism include COG0524 (sugar kinase family), COG2814 (major facilitator superfamily), COG1263 (phosphotransferase system), COG0477 (major facilitator superfamily), COG1940 (glucokinase), and COG2723 (glycoside hydrolase family 1). These genes participate in critical processes such as carbohydrate transport, phosphorylation, and glycosidic bond hydrolysis, collectively forming a complete carbohydrate metabolic pathway that provides a solid genetic foundation for the strain’s cellulose-degrading capability [19,20].

In GO annotation, 137 genes associated with carbohydrate metabolism (GO: 0005975) and 40 genes associated with glycoside hydrolase activity (GO: 0016798) further corroborate the strain’s functional advantages in carbohydrate degradation from the perspectives of molecular function and biological process. KEGG pathway annotation results showed that a total of 4203 genes in the *B. subtilis* A2 genome were assigned annotations, distributed across six major pathway categories: cellular processes, environmental information processing, genetic information processing, human diseases, metabolism, and organismal systems. Among these, metabolism-related genes were the most abundant (1403 genes), accounting for 33.38% of the total annotated genes, indicating that metabolic processes constitute the core physiological activity of this strain. Within the metabolism-related genes, those involved in carbohydrate metabolic pathways numbered 413, representing 29.44% of the total metabolic genes. Among these, significant enrichment was observed in pathways such as sucrose and starch metabolism (ko00500, 46 genes), glycolysis/gluconeogenesis (ko00010, 38 genes), pyruvate metabolism (ko00620, 43 genes), and the pentose phosphate pathway (ko00030, 26 genes). These pathways constitute a complete network for carbohydrate degradation and conversion: complex carbohydrates such as cellulose are first degraded into monosaccharides like glucose, which are subsequently converted into energy or metabolic intermediates through pathways such as glycolysis and pyruvate metabolism, supporting the strain’s growth, reproduction, and synthesis of secondary metabolites. Additionally, the presence of genes related to energy metabolism (133 genes) and glycan biosynthesis and metabolism (31 genes) further refines the strain’s metabolic network, enabling it to efficiently utilize various carbon sources and adapt to diverse environmental conditions.

### 4.3. CAZyme Families and the Lignocellulose Degradation Mechanism: The Scientific Connotation of Synergy

Systematic annotation of carbohydrate-active enzymes (CAZymes) reveals the molecular mechanism underlying the efficient lignocellulose degradation by *B. subtilis* A2. The 167 CAZyme genes account for 4.09% of the total CDS, a proportion significantly higher than that of ordinary *B. subtilis* strains (average proportion of 3.2%) [21,22]. Among these, the enrichment of 64 genes from glycoside hydrolase (GH) families constitutes the core force for cellulose degradation. The four genes from the GH1 family encode β-glucosidases, which effectively relieve product inhibition of cellulases by cellobiose, a critical factor in enhancing cellulose degradation efficiency [23,24]; the four genes from the GH3 family encode exo-β-1,4-glucanases, which cleave cellobiose from the non-reducing ends of cellulose chains; the five genes from the GH4 family encode 6-phospho-β-glucosidases, involved in the metabolism of phosphorylated cellobiose. Together, these form a complete cellulolytic enzyme system comprising “endoglucanase-exoglucanase-β-glucosidase.”

More importantly, the A2 strain’s genome encodes 60 hemicellulases and three lignin-degrading enzymes, establishing a synergistic and efficient lignocellulose degradation system. Among the hemicellulases, 24 acetyl xylan esterases (including 11 from the CE1 family) hydrolyze acetyl side chains on hemicellulose molecules, reducing steric hindrance of the substrate; 11 feruloyl esterases (from the CE1 and CE7 families) cleave the ester bonds between ferulic acid and xylan, disrupting the cross-linking structure between hemicellulose and lignin [18,25]; β-xylanases (GH11 family) and β-mannosidases (GH26 family) specifically degrade the main chain structure of hemicellulose. The synergistic action of these enzymes enhances hemicellulose degradation efficiency by over 35% [26,27]. Regarding lignin degradation, laccases and multicopper oxidases from the AA1 family disrupt the aromatic ring structure of lignin through redox reactions, reducing its encapsulation of cellulose and hemicellulose. This characteristic enables the A2 strain to achieve a total saccharification efficiency 28–32% higher than that of strains capable of degrading only cellulose when utilizing natural lignocellulosic substrates (such as corn stover and wheat bran) [28,29]. Furthermore, the presence of 17 carbohydrate-binding module (CBM) genes enhances enzyme-substrate binding affinity, further improving degradation efficiency. This “enzyme-module-substrate” binding mechanism represents a crucial guarantee for the A2 strain’s efficient degradation of complex substrates [30,31].

*B. subtilis* A2 simultaneously possesses enzyme genes related to cellulose, hemicellulose, and lignin degradation, forming a complete lignocellulolytic enzyme system. This characteristic enables *B. subtilis* A2 to independently accomplish the degradation of all three major components of lignocellulose, significantly enhancing its application value in the utilization of lignocellulosic resources and holding promise for simplifying fermentation processes and reducing production costs.

### 4.4. Enzymatic Properties of Cellulase: Key Support for Industrial Application Potential

Quantitative analysis of enzymatic properties is central to evaluating the application value of cellulases. All indicators of the *B. subtilis* A2 cellulase demonstrate significant industrial suitability. Its optimal temperature of 55 °C enables it to retain over 85% of its enzymatic activity during feed pelleting (typically conducted at 55–60 °C for 10–15 min), whereas the residual activity of ordinary *B. subtilis* cellulase under the same temperature conditions is only 60–70% [32,33]. The optimal pH of 7.0 is highly compatible with the neutral to slightly alkaline environment of the livestock and poultry intestine. Compared with cellulases that have an acidic optimal pH (such as those derived from *Aspergillus niger*, with an optimal pH of 4.8), the A2 strain’s enzyme exhibits a reduction in activity loss within the intestine by over 40% [34,35]. In thermal stability experiments, the enzyme retained 60% of its activity after incubation at 55 °C for 2 h. Regarding pH stability, enzyme activity remained above 80% after 18 h of storage in the pH range of 5–7, providing favorable conditions for long-term storage of the enzyme preparation. The cellulase from strain A2 showed optimal activity at 55 °C and pH 7.0, and its degradation efficiency toward microcrystalline cellulose reached 197% relative to CMC-Na. These properties are superior to many reported Bacillus subtilis cellulases, which usually show lower activity on crystalline cellulose and poorer thermal stability. Compared with commercial feed-use cellulase products, strain A2 cellulase has better adaptability to neutral pH and intestinal temperature, making it more suitable for application in poultry feed.

The results of the metal ion influence experiment offer important practical guidance: 1 mmol/L Cu^2+^ led to a 59.2% decrease in enzyme activity. This inhibitory effect may be associated with the oxidative modification of serine residues in the enzyme’s active center or the spatial disruption of the catalytic domain [36]. The inhibition rates for Mn^2+^ and Zn^2+^ were 46.6% and 46.1%, respectively, which is speculated to be related to the competition between these metal ions and the enzyme for metal-binding sites in the active center. In practical applications, the inhibitory effect can be mitigated through two strategies: first, employing ion-exchange chromatography to remove metal ion impurities during enzyme preparation production; second, adding chelating agents such as EDTA (at a concentration of 0.05–0.1%) to feed formulations to chelate metal ions and thus reduce their impact on enzyme activity [37,38].

Substrate specificity analysis revealed that the A2 strain’s enzyme exhibited a relative enzyme activity of up to 197% towards microcrystalline cellulose. This result indicates that the A2 enzyme possesses a strong capacity for deconstructing crystalline cellulose—likely through a unique catalytic mechanism, such as the synergistic action of glycoside hydrolase and carbohydrate-binding modules (CBMs)—to disrupt the hydrogen bond network of cellulose. This characteristic precisely addresses the shortcoming of existing commercial enzyme preparations, which exhibit low degradation efficiency towards crystalline cellulose [39,40]. Furthermore, the enzyme’s degradation activity towards wheat bran and absorbent cotton (with relative enzyme activities of 48.4% and 49.2%, respectively) positions it with broad application prospects in feed fermentation or biomass energy conversion utilizing agricultural by-products as raw materials.

## 5. Conclusions

In summary, this study successfully isolated a highly efficient cellulose-degrading bacterium, *B. subtilis* A2, from the intestine of Yangzhou geese. The intestinal origin suggests that this strain and its enzyme are particularly well-adapted for use as a feed supplement in poultry, as the optimal pH (7.0) align with the neutral pH and temperature conditions of the animal’s digestive tract. Beyond direct application in animal nutrition, the A2 cellulase’s remarkable ability to degrade microcrystalline cellulose (197% relative activity) points to broader industrial potential, including the bioconversion of lignocellulosic biomass for bioenergy and biochemical production. The comprehensive CAZyme families and carbohydrate metabolic pathways identified through whole-genome sequencing provide the genetic foundation for its efficient lignocellulose degradation. Our findings, therefore, offer a valuable microbial strain for the development of targeted poultry feed enzymes and a robust genetic resource for advancing lignocellulose bioconversion technologies.

## Figures and Tables

**Table 1:** Hydrolysis circle ratio (HCR) of isolated strains on CMC agar medium.

Strains	D/mm	d/mm	HCR (D/d)
A1	1.46 ± 0.08	1.31 ± 0.04	1.11 ^cd^ ± 0.03
A2	3.44 ± 0.02	2.32 ± 0.03	1.48 ^a^ ± 0.03
A3	4.30 ± 0.29	4.11 ± 0.04	1.05 ^d^ ± 0.07
A4	4.37 ± 0.03	3.97 ± 0.02	1.10 ^cd^ ± 0.01
B1	1.48 ± 0.04	1.28 ± 0.05	1.16 ^bc^ ± 0.02
B2	2.94 ± 0.02	2.55 ± 0.20	1.16 ^bc^ ± 0.09
B3	4.45 ± 0.03	4.17 ± 0.03	1.07 ^d^ ± 0.01
B4	5.91 ± 0.21	4.92 ± 0.22	1.20 ^b^ ± 0.01

Values are expressed as mean ± SD (n = 6). Different lowercase letters mean the significance at *p* < 0.05. D, The diameter of the hydrolysis circle; d, The diameter of the colony; HCR (D/d value), The hydrolysis ratio of the strain.

**Table 2:** Basic features of strain A2 genome.

Property	Value	Percent of Genome (%)
Total sequence length	4,021,044 bp	/
GC content	/	43.72
CDS	4083	/
The total length CDS	3,561,132 bp	88.56
The number of tRNA	59	0.1132
The number of ncRNA	93	0.3681
The number of Prophages	18	/
The number of CRISPRs	1	/

CDS, protein coding genes.

**Table 3:** OrthoANIu and dDDH values between strain A2 and closely related type strains.

Tape Strain	Reference Genome	OrthoANIu (%)	dDDH (%)
*Bacillus subtilis* ATCC 6051	GCF_000009045.1	99.06	92
*Micromonospora provocatoris* MT25	GCF_003298855.1	93.47	83
*Bacillus stercoris* D7XPN1	GCF_000738015.1	95.31	62.3
*Bacillus spizizenii* TU-B-10	GCF_000227465.1	93.08	50.4
*Bacillus inaquosorum* KCTC 13429	GCF_000332645.1	93.04	50
*Bacillus rugosus* SPB7	GCF_011745685.2	92.77	48.8
*Bacillus cabrialesii* TE3	GCF_004124315.2	92.37	47.7
*Bacillus cabrialesii* subsp. *tritici* TSO2	GCF_018390475.2	92.45	47.6
*Bacillus tequilensis* NCTC13306	GCF_000507145.1	91.72	45.3
*Bacillus vallismortis* DV1-F-3	GCF_000245315.1	90.95	42.4
*Bacillus mexicanus* FSQ1	GCF_020223575.1	90.22	39.7

Note: Species thresholds: OrthoANIu ≥ 95–96%, dDDH ≥ 70%.

**Table 4:** Annotated genes encoding lignocellulose-degrading enzymes in *B. subtilis* A2.

	Gene ID	Genes Count	CAZy	Enzymes
Cellulose-related	s2-1240; s2-1327; s4-3373; s4-3619	4	GH1	β-glucosidase (EC 3.2.1.21)
	s4-3202	1	GH3	β-glucosidase (EC 3.2.1.21)
	s2-1240; s2-1327; s4-3373; s4-3619	4	GH1	exo-β-1,4-glucanase (EC 3.2.1.74)
	s4-3202	1	GH3	exo-β-1,4-glucanase (EC 3.2.1.74)
	s1-9; s1-853; s2-1397; s5-3681; s5-3780	5	GH4	6-phospho-beta-glucosidase (EC 3.2.1.86)
	s2-1240; s2-1327; s4-3373; s4-3619	4	GH1	6-phospho-beta-glucosidase (EC 3.2.1.86)
	s4-3202	1	GH3	exo-1,3-1,4-glucanase (EC 3.2.1.-)
Hemicellulose-related	s1-1071	1	GH11	endo-β-1,4-xylanase (EC 3.2.1.8)
	s2-1240; s2-1327; s4-3373; s4-3619	4	GH1	beta-xylosidase (EC 3.2.1.37)
	s4-3202	1	GH3	xylan 1,4-beta-xylosidase (EC 3.2.1.37)
	s1-474; s4-3623	2	GH26	beta-mannanase (EC 3.2.1.78)
	s2-1240; s2-1327; s4-3373; s4-3619;	4	GH1	beta-mannosidase (EC 3.2.1.25)
	s4-3202	1	GH3	alpha-L-arabinofuranosidase (EC 3.2.1.55)
	s1-69; s1-853; s2-1397; s5-3681; s5-3780	5	GH4	alpha-glucuronidase (EC 3.2.1.139)
	s1-69; s1-853; s2-1397; s5-3681; s5-3780	5	GH4	alpha-galactosidase (EC 3.2.1.22)
	s1-1001; s1-19; s1-41; s1-431; s2-1907; s2-2076; s3-2430; s3-2505; s3-3196; s3-3640; s3-3963	11	CE1	feruloyl esterase (EC 3.1.1.73)
	s1-1001; s1-19; s1-41; s1-431; s2-1907; s2-2076; s3-2430; s3-2505; s4-3196; s5-3640; s6-3963	11	CE1	acetyl xylan esterase (EC 3.1.1.72)
	s1-547; s2-1341; s5-3786; s5-3791	4	CE12	acetyl xylan esterase (EC 3.1.1.72)
	s1-964	1	CE3	acetyl xylan esterase (EC 3.1.1.72)
	s2-1373; s3-2277; s3-2534; s3-3014; s5-3702; s6-3919	6	CE4	acetyl xylan esterase (EC 3.1.1.72)
	s2-2055; s4-3347	2	CE7	acetyl xylan esterase (EC 3.1.1.72)
	s2-1866	1	GH53	endo-beta-1,4-galactanase (EC 3.2.1.89)
	s1-1071	1	GH11	exo-1,4-beta-xylosidase (EC 3.2.1.-)
Lignin-related	s5-3872	1	AA1	Laccase (EC 1.10.3.2)
	s5-3872	1	AA1	ferroxidase (EC 1.10.3.-)
	s5-3872	1	AA1	Laccase-like multicopper oxidase (EC 1.10.3.-)

## Data Availability

The original contributions presented in this study are included in the article/Supplementary Material. Further inquiries can be directed to the corresponding author.

## Supplementary Materials

The following supporting information can be downloaded at: https://www.mdpi.com/article/10.3390/microorganisms14061272/s1, Supplementary Table S1: COG functional classification of *B. subtilis* A2 genome; Supplementary Table S2: COG annotation of *B. subtilis* A2 genome; Supplementary Table S3: GO annotation of *B. subtilis* A2 genome; Supplementary Table S4: GO annotation of *B. subtilis* A2 genome; Supplementary Table S5: KEGG annotation of *B. subtilis* A2 genome; Supplementary Table S6: Functional annotation of CAZy databases of *B. subtilis* A2.
